# The CD4:CD8 ratio is associated with IMT progression in HIV-infected patients on antiretroviral treatment

**DOI:** 10.7448/IAS.17.4.19723

**Published:** 2014-11-02

**Authors:** Enrique Bernal, Jose Serrano, Ana Perez, Salvador Valero, Eva Garcia, Irene Marín, Angeles Muñoz, Jose Miguel Gomez Verdú, Carmen Vera, Alfredo Cano

**Affiliations:** 1 Reina Sofia Hospital, Infectious Disease Unit, Murcia, Spain; 2 Reina Sofia Hospital, Cardiology, Murcia, Spain

## Abstract

**Introduction:**

Inversion of the CD4:CD8 ratio (<1) has been identified as a hallmark of immunosenescence and an independent predictor of mortality in the general population. We aimed to assess the association between the CD4:CD8 ratio and intima-media thickness (IMT) progression in treated HIV-infected patients as a marker of early atherosclerosis.

**Materials and Methods:**

A longitudinal study during three years was conducted in 120 HIV-infected patients receiving antiretroviral treatment (ART). We analyzed the associations between the CD4:CD8 ratio, cardiovascular risk factor and antiretroviral (ARV) treatment and progression of subclinical atherosclerosis assessed using carotid IMT at baseline and after three years.

**Results:**

Finally, 96 patients completed the study. Seventy-six (79.1%) patients were male, aged 44±10 years, 39 (40.6%) were on treatment with Protease inhibitors, 49 (51.04%) with non-nucleoside reverse transcriptase inhibitors (NNRTI), 6 (6.25%) with integrase inhibitors, 3 (3.12%) with maraviroc and 2 (2.08%) only with nucleoside reverse transcriptase inhibitors (NRTI). The mean of ARV exposition was 6.9±5.9 years. Twenty six (27 %) patients had family history of ischemic heart disease, 51 (53.12%) were smokers, 12 (12.5%) hypertensive, 4 (4.16%) type 2 diabetes, 23 (23.9%) with dyslipidemia and 31 (32.3%) were infected with C hepatitis virus. Baseline IMT was significantly associated with age (rho=0.497; p<0.001), basal glucemia (rho=0.323; p=0.001), triglycerides (rho=0.232; p=0.023), Framingham score (rho=0.324; p=0.001), CD4:CD8 ratio (rho=−0.176; p=0.05) and dyslipidemia (0.72±0.16 mm vs 0.63±0.11 mm; p=0.029). In multivariable analysis where cardiovascular risk factor and ARV were included, IMT progression was inversely associated with CD4:CD8 ratio (OR=0.283; CI 95% 0.099–0.809; p=0.019) and treatment with NNRTI (OR=0.283; CI 95% 0.099–0.809; p=0.019).

**Conclusions:**

The inversion of CD4:CD8 ratio in treated HIV-infected patients is independently associated with IMT progression, a marker of age-associated disease. Therefore, it might be clinically useful as predictor of cardiovascular events. Surprisingly, there was a positive correlation between receiving NNRTI and progression of IMT.

